# Food Leftover Practices among Consumers in Selected Countries in Europe, South and North America

**DOI:** 10.3390/foods5030066

**Published:** 2016-09-21

**Authors:** Kadri Koppel, Federica Higa, Sandria Godwin, Nelson Gutierrez, Roman Shalimov, Paula Cardinal, Brizio Di Donfrancesco, Miriam Sosa, Angel A. Carbonell-Barrachina, Loreida Timberg, Edgar Chambers

**Affiliations:** 1 The Sensory Analysis Center, Kansas State University, Manhattan, KS 66503, USA; higa@ksu.edu (F.H.); briziod@ksu.edu (B.D.D.); eciv@ksu.edu (E.C.IV); 2 Department of Family and Consumer Services, Tennessee State University, Nashville, TN 37209, USA; sgodwin@tnstate.edu; 3 Centro Surcolombiano de Investigación en Café (CESURCAFE), Facultad de Ingeniería, Universidad Surcolombiana, Neiva 660003, Colombia; ngutierrezg@usco.edu.co; 4 Tochka Rosta, 1 Kvesisskaya str., 18, Trade Center Bld, Moscow 127220, Russia; rs@tochkarosta.ru; 5 Comisión de Investigaciones Científicas Provincia de Buenos Aires (CIC), Instituto Superior Experimental de Tecnología Alimentaria (ISETA), 9 de Julio, Buenos Aires 6500, Argentina; paula@desa.edu.ar; 6 Consejo Nacional de Investigaciones Científicas y Técnicas (CONICET), Instituto Superior Experimental de Tecnología Alimentaria (ISETA), 9 de Julio, Buenos Aires 6500, Argentina; miriam@desa.edu.ar; 7 Departamento Tecnología Agroalimentaria, Escuela Politécnica Superior de Orihuela, Universidad Miguel Hernández de Elche, Orihuela 03312, Alicante, Spain; angel.carbonell@umh.es; 8 Department of Food Processing, Faculty of Chemical and Materials Technology, Tallinn University of Technology, Ehitajate tee 5, Tallinn 19086, Estonia; loreida.timberg@ttu.ee

**Keywords:** consumer, cross-country, food safety, leftovers

## Abstract

Foodborne illnesses may be related to many food production factors with home practices of consumers playing an important role in food safety. Consumer behavior for handling food leftovers has been studied, however little work on comparisons among countries has been published. The objective of this study was to investigate home food leftover practices of people from North American, South American, and European countries. Surveys were conducted with approximately 100 or more consumers in Argentina, Colombia, the United States, Estonia, Italy, Russia, and Spain. The participants responded to questions related to the length of time different types of food leftovers; such as meat, fresh salads, or restaurant dishes would be kept refrigerated or would be left at room temperature before refrigeration. Researchers also investigated how consumers would determine if the food was still safe for consumption. Potentially risky behaviors were observed in all seven countries. For instance, 55.8% of Estonians, 25% of Russians and 25.8% of Argentinean participants left food out at room temperature for several hours before storing in the refrigerator. Furthermore, 25%–29% of Colombian, Estonian, and Spanish consumers would look, smell, and taste leftovers to determine its probable safety. Correct handling of leftovers is an important aspect of consumer food safety. Although the surveys cannot be representative of all consumers in each country, they do provide an initial overview of comparative practices for handling leftovers among different countries. This provides government and educators with information on potential universal and unique consumer food safety issues related to handling leftover foods among various countries.

## 1. Introduction

Poor food handling may result in a large number of foodborne illnesses in many countries [1]. Although food handling risks can be reduced during production, the consumer has the ultimate responsibility for the safe storage and preparation of food eaten in the home. To reduce foodborne illnesses, consumers need to implement proper food handling practices. Moreover, a large portion of consumers underestimate or are unaware of the risk of contracting foodborne diseases from improper practices related to handling food at home [2].

Food safety guidelines are abundant in the United States [3], but less often in other countries. There are general food safety guidelines available by the World Health Organization that have been translated into several languages [4]. However, it is not clear how widespread the knowledge of these guidelines are by consumers. Government and educational websites in many countries do not mention the availability of educational food safety programming to consumers and little data has been published showing widespread adoption and use of the guidelines in health campaigns. Contrary to food companies that must follow legislative requirements, consumers are not supervised when handling food and have to make decisions based on prior knowledge, experience, convenience, and practicalities. This can constitute a public health problem, especially when cross-contamination involves pathogens such as *Escherichia coli*, *Salmonella*, or *Campylobacter*. In Europe and North America, more than half of the registered food infections are contracted at home [1]. Pathogenic bacteria have been found in home-cooked meals [5] and can grow rapidly even if food is stored following proper temperature guidelines [6].

Some of the issues with food leftovers that may result in foodborne illnesses include using containers that the food was cooked or served in to store leftovers, the time and storage conditions used for leftover foods, and the initial cooking and reheating processes used. Bruhn and Shutz indicated that half of the participants in their survey sometimes stored food in large containers where the food was prepared, while only two-thirds reported using smaller containers for storage, which is the recommended practice [7]. Furthermore, consumer sociodemographic backgrounds may have an influence as well. For example, males and young participants felt less responsibility for their own safety when preparing food than women or older people [2]. Marklinder et al. also found differences between older and younger participants when it came to the amount of days leftovers were stored, showing that older people tended to save food for fewer days [1]. Kwon et al. (2008) and Terpstra et al. (2005) observed older consumers and found that practices were different than those of younger participants [8,9]. In addition, Cody et al. found that women stored leftover food better than men [10]. Furthermore, Kwon et al. found that education level had an influence when deciding the safest way to store leftovers [8].

Cody et al. reported that several food safety practices, including storing leftovers correctly, declined from 2008–2010 in the United States [10]. This indicates that consumers need to be continuously informed about proper food-handling practices. Some studies have compared food safety practices across countries. For example, egg- and poultry-related practices were studied in Asian countries [11], in European countries [12], and in North and South America [13]. No studies were found that investigated how leftover food practices differ among countries. This type of information may be useful when developing food safety-related consumer messages around the world. Various pan-governmental and educational organizations develop and promote guidelines for food safety that transcend countries, but have little actual comparative data from a single country to rely on; particularly when it comes to consumer food-safety issues.

The objective of this study was to understand consumer behavior towards food leftover storage in selected countries from North and South America (Argentina, Colombia and the United States) and Europe (Estonia, Italy, Russia and Spain), particularly as it is related to:

(1) times various foods might be left out before storage as leftovers;
(2) times various leftover foods typically are stored in the refrigerator; and
(3) how consumers determine if leftover foods are still good to eat.

## 2. Materials and Methods

### 2.1. Questionnaires and Data Collection

The questionnaires were originally created in English, then translated into Italian, Estonian, Russian, and Spanish by native speakers, and back-translated into English to ensure the information the questionnaires were gathering matched across countries. The questionnaires were answered by participants either online or at a central location, using paper surveys that were handed to the consumers to be completed.

Questions concerning demographic information such as: gender, age (≥35 and <35 years old), educational level (high school education or less; some college education or a higher degree), and household income (categories equivalent as determined by each country (low: <$25,000; medium: $25,000–$50,000; high: >$50,000) and were included at the end of the questionnaire (Table 1).

Questions regarding leftover storage investigated how leftover foods were stored when a meal is cooked in the household; food storage frequency; time food is left out at room temperature before being refrigerated; containers used to store leftovers in refrigeration and time certain leftovers (meat dishes, vegetables, fresh salad, cooked pasta, desserts, fruits, restaurant dishes) could be stored before spoilage occurred; and how leftover safety was determined by the consumers.

### 2.2. Respondents

About 100 participants in each country were surveyed and they were selected according to a pre-screening survey. The consumers were over 18 years old; involved in food shopping, cooking, and storing food in the households—primary or shared responsibility for shopping, cooking, and storage—and they had to own a refrigerator for food storage. Similar criteria has been used in studies by Koppel et al. that investigated Asian consumer food safety practices [11] and European consumer food safety practices [12].

Respondents (Table 1) completed the questionnaire from the United States (from the states of Kansas, Missouri and Tennessee), Argentina (Buenos Aires province), Colombia (Huila, Neiva region), Italy (Lecce area), Estonia (Tallinn area), Russia (Moscow area), and participants from Spain (Alicante area). Most consumers were recruited from established consumer databases (USA—Kansas and Missouri—Argentina, Estonia, and Spain) or mall intercept (Russia); but some consumers were a convenience sampling from the local area (USA: Tennessee—parents of local school children, faculty, staff, students at two local colleges; Colombia—homemakers and office workers; Italy—local e-mail server and snowball sampling or word of mouth). Consumers were divided by age (<35 and ≥35) because other studies of food safety behaviors have found differences in younger and older consumers [1,2,8,9]. It should be noted that the studies, although conducted predominantly by university researchers, did not target students and attempted to avoid student areas or populations during recruiting. The limited number of consumer sampling procedures did not cover each category of the population, but provides an initial insight into the behavior of a general population for leftover foods in each country.

### 2.3. Data Analysis

Data analysis was performed using frequencies, percentages, and the CHITEST Excel function (Microsoft Excel 2010; Microsoft, Redmond, WA, USA) to study differences among countries and differences based on sociodemographic information within and among countries.

## 3. Results

### 3.1. Consumer Demographics

The respondents represented a mixture of genders, ages, education, and income levels (Table 1). In most countries studied, a mixture of males and females were recruited; however in Argentina, all of the respondents were female. In addition; in Estonia, Italy, and Russia, ≤16% of respondents were male. Although this may be because of sampling, this more likely is because of food purchasing, preparation, and storage behaviors (the qualifying criteria) in those countries typically done by females. Thus, the low percentage of males completing the survey in those countries is not surprising. The age distribution of the respondents was not evenly distributed among countries; particularly in Estonia, where the percentage of people in the survey is heavily skewed towards over 35 and clearly appears to have been impacted by the sampling procedure. To the extent that age impacts decisions on leftovers can be a confounding affect with countries in this study. Education levels among the respondents was higher (some college or more) in the United States, Italy, Estonia, Russia, and Spain than in other countries; in part reflecting the country; in part reflecting the communities in which the surveys were conducted, and in part reflecting the differences in survey methodology used in various locations. Income of the respondents was distributed over the three categories in most countries; although in Colombia, the majority of the respondents declared their income as “low” and in Estonia, most respondents declared their income as “low” or “medium”—both most likely related to the in-country decisions on what constituted a low, medium, or high income compared to the dollars set for the United States.

### 3.2. Behavior of Saving Leftovers

**Figure 1:**
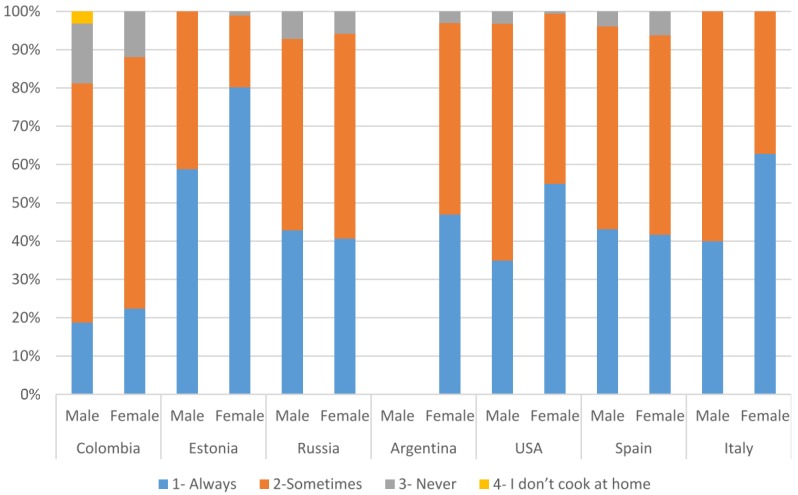
When you cook at home, do you usually save your leftovers? Results presented for women and men in each country, by percentage.

The participants were asked if and how often they usually saved leftovers (Table 2). In all countries, most participants (>80%) saved leftovers always or sometimes (Table 2). Only 13.1% of Colombian, 5.1% of Spanish, and 6% of Russian participants indicated they never saved leftovers. Women tended to save leftovers more frequently than men, except for Russia and Spain, where the behavior for saving food was similar for both genders (Figure 1). This may be caused by several factors, such as men cooking less than women, or perhaps women being responsible for saving leftovers after a meal.

**Figure 2:**
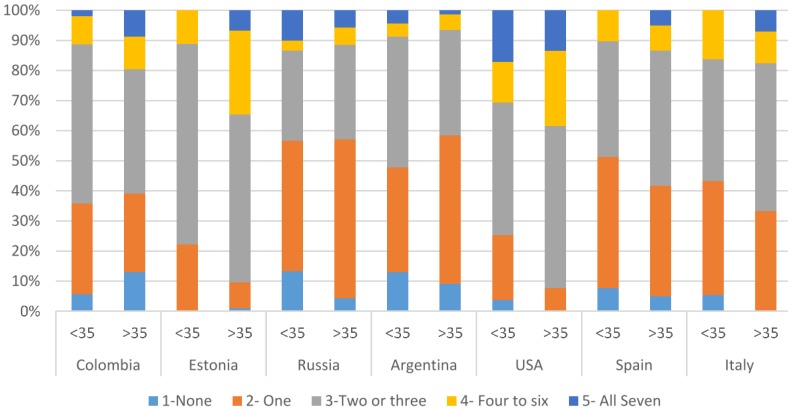
If someone were to look in your refrigerator each day for a week, on how many of the seven days do you think they would find leftover food in your refrigerator? Results presented for respondents aged <35 and ≥35 in each country, by percentage.

Almost half of participants saved leftovers with a frequency of two to seven days per week (Table 3). Participants in Russia and Argentina were an exception, where 57% and 56% of the participants respectively saved food leftovers only once a week or never (Table 3). Estonian and the United States respondents tended to save their leftovers more frequently. Marklinder et al. found that older Swedish participants tended to save foods for shorter days than did younger participants [1]. This was not necessarily the case in this study, however. In most of the countries studied, it was found that younger and older people tended to save leftovers in a similar fashion, although the proportion of older respondents who saved leftovers for four or more days was larger in Colombia, Estonia, and the United States (Figure 2).

### 3.3. Time Food is at Room Temperature

Participants were asked how long leftovers were left out before storing them in the refrigerator after cooking for a holiday meal. A holiday meal was chosen specifically for this study because it represents a time when many people gather and people may tend to leave food out for snacking over the course of the day. Also, more people may eat the leftovers at a later time, resulting in a greater potential for foodborne illness. About half of the participants in the United States, Spain, Russia, Italy, and Argentina indicated that they let food sit at room temperature between half an hour and an hour (Table 4). In Colombia, 39.4% of participants left food out for up to two hours before storing it in the refrigerator.

**Figure 3:**
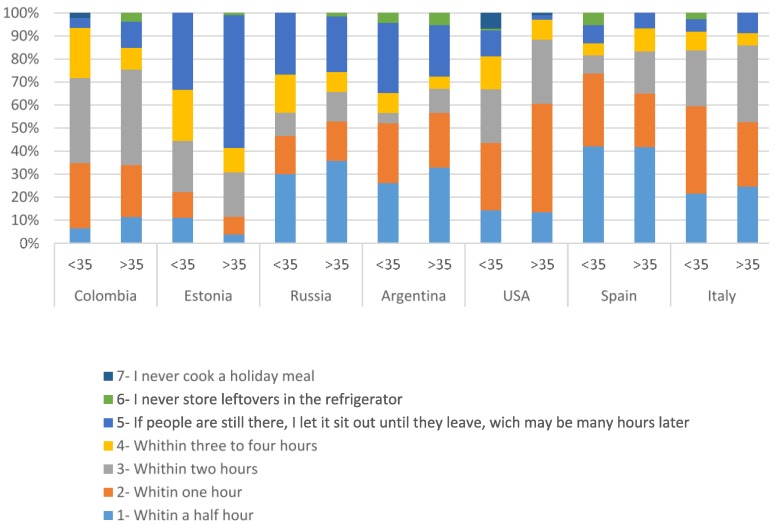
You have prepared food for a holiday meal and there is food left over. How long do you generally let it sit out at room temperature before putting it in the refrigerator? (Select one.) Results presented for respondents aged <35 and ≥35 in each country, by percentage.

In the United States, most people older than 35 (60.6%, Figure 3) stored food within an hour from preparation; while for Argentina, 30.4% of participants younger than 35 years old left food out for several hours. Both in Argentina and Colombia, the majority of participants over 35 did not leave food out for periods longer than two hours (Figure 3).

Italy, Spain, and Russia showed similar results to those in North and South American countries, where the majority stored food within one hour. On the other hand, for Estonian participants, 55.8% indicated that they left food out for longer periods of time, which may be many hours after the meal was prepared. The majority of these respondents were participants over 35 years old (Figure 3). A similar response was selected by 24.8% of Argentinian and 25% of Russian respondents.

Italian participants, with less than college education, saved food within one hour (43.7%); and 31.6% of people, with some college education, saved leftovers within two hours. However, 12.5% of Italian respondents, with less than college education, let food sit out for several hours; while 7.6% of people, with some college education, did the same thing. In Russia, most participants saved food within half an hour (34%); but people with college education left food out for longer periods (27.7%) than people with less than college education (11.7%).

By income, 40.9% of high-income Italian consumers had food at room temperature for two hours, while medium- and the majority of low-income respondents left food out for an hour from preparation. According to several studies, young adults take more risks when it comes to food safety, such as eating raw foods of animal origin [8,14,15,16]. On the other hand, Terpstra et al. indicated that older people lacked knowledge about how long food should be stored [9]. Altekreuse also reported that men and people with higher socioeconomic status tend to take more risks [16], while Klontz reported the same behavior for participants with an education lower than high school level [14]. The results from this study indicated that sociodemographic indicators may not predict behavior in a similar way in different countries.

### 3.4. Containers for Storing Leftovers

Participants were asked about the way they usually saved food leftovers. Participants from the United States and Italy stored leftover food mainly using special containers, and plastic or aluminum wrap as a second choice. Similarly, the majority of Estonian participants indicated the use of a special container to store food as the most popular option, but as the second option; they indicated using the container in which the food was cooked in (Table 5). The majority of people with high incomes (55.5%) chose plastic or aluminum foil as a first option, while medium or low incomes preferred special containers or the container in which food was served in.

The majority of Argentinian participants (60.9%) saved food in special containers (Table 5). For participants under 35, the second most popular storage was to save food in the container in which food was served (17.85%), while people over 35 preferred to save leftover food in plastic or aluminum foil (21%). As a second choice, people with less than a college education (16.9%) and people with low incomes (19.7%) indicated that they saved food in the container which food was served, while people with higher education level (21.1%)—as well as with medium (20%) and high (21.9%) incomes—indicated using plastic or aluminum foil to store leftovers.

**Figure 4:**
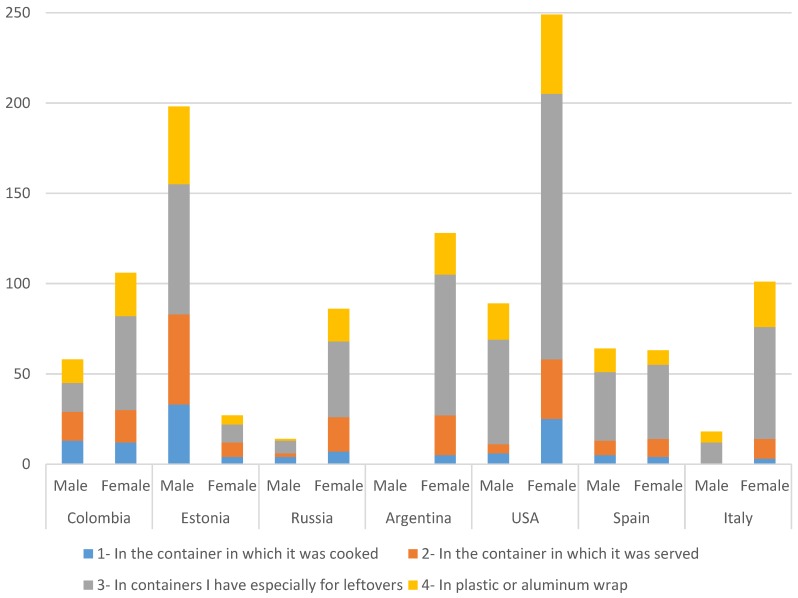
How do you usually store leftover food in the refrigerator? (Check all that apply). Results presented for males and female in each country, by count.

Similarly, in Russia, the most frequent option was to use special containers for all respondents. However, a second frequent option for males was using the container which the food was cooked (28.6%), while females (20.9%) preferred plastic or aluminum foil. In Colombia, males and females indicated that they handled leftovers differently. While most of the women (49.1%) chose to use containers specially designed to store food, men had no preference (Figure 4).

Participants in Spain indicated they use special containers as the most frequent option for food storage, but the second most frequent option varied between sociodemographic groups. Males (20.3%), participants over 35 (15.3%), and high-income participants (21.1%) chose plastic or aluminum foil, while females (16.94%), participants under 35 (20%), and low- and medium-income participants (23.3% and 18.9%) chose using the container which the food was served.

### 3.5. Time Food Can Be Stored before Spoiling

Respondents were asked how long they thought certain leftovers; such as meat dishes, vegetable dishes, fresh salads, cooked pasta, desserts, fruits, and restaurant dishes can stay in the refrigerator before spoiling. The results varied quite a bit depending on the food category.

#### 3.5.1. Meat Dishes

Terpstra et al. found that although most of the consumers in their study perceived meat a risky product when considering storage time, about half of them stored meat dishes for a longer period than the one recommended in the guidelines [9]. The United States Food Safety and Inspection Service (FSIS) guidelines suggest storing leftovers for a maximum of 3–4 days in the refrigerator [17]. Most participants in this study indicated that they thought meat dishes can be stored for 3–4 days in the refrigerator (Table 6). A total of 12.4% of the United States, 5.1% Colombian, 5% of Argentinian, and 5% of Russian respondents indicated they thought that meat dishes can be stored for up to a week.

#### 3.5.2. Vegetables, Fresh Salad, Fruit

Most of the respondents indicated 1–2 days as a safe storage time for vegetables, while 17.6% of the United States participants indicated storing vegetable leftovers for up to a week (Table 6). The safe practices for vegetable storage vary by the source and quality of the vegetables. In comparison to meat dishes, it is perhaps somewhat easier to determine whether vegetables have deteriorated in quality. The majority of the participants indicated that fresh salads could be stored for 1–2 days before spoiling would occur (Table 6). Most of the respondents from all countries indicated that one week was the period of time that fruit could be stored before spoiling.

#### 3.5.3. Pasta

The majority of the participants indicated that pasta could be stored for 1–4 days before spoiling would occur (Table 6). However, a total of 29% of the United States and 17.7% of Estonian participants responded that they would store cooked pasta for up to a week. Whether this practice is risky or not, depends somewhat on the type of pasta dish. For pasta dishes with sauces, the safe storage time may be shorter; while for pasta alone, this may not be true. Aside from the storage time, the consumers should also follow safe reheating guidelines [17].

#### 3.5.4. Desserts

In most of the countries, participants indicated that desserts could be stored up to four days before spoiling, except for the United States, where 42.2% of respondents stated that this type of food can be stored for up to a week (Table 6). Depending on the dessert ingredients and cooking method, this may either be considered a safe or a risky behavior.

#### 3.5.5. Restaurant Dishes

The majority of the consumers indicated that restaurant dish leftovers are safe to store for up to four days in the refrigerator. This question elicited a large percentage of “do not know” responses. Of all Argentinian, Russian, Estonian and Italian participants, 32%, 26%, 15%, and 23.9% respectively, indicated not knowing how long to store restaurant dish leftovers (Table 6). More than 30% of Russian participants with college education and 44% of less than college participants in Estonia indicated not knowing how many days restaurant dishes could be stored before spoiling. Perhaps this may also be associated with the frequency the participants in that country are used to eating out at restaurants. In some countries, such as the United States, eating out at restaurants and bringing home leftovers from that meal may be a more common practice than in others.

A total of 32% of Argentinian participants did not know how long restaurant dishes could be stored before spoiling (Table 6). This segment of consumers included 38.9% of people over 35 years old, 39% with less than college education, 36.5% with low income and 33.3% with medium income.

### 3.6. Determining Leftovers’ Safety in the Refrigerator

**Figure 5:**
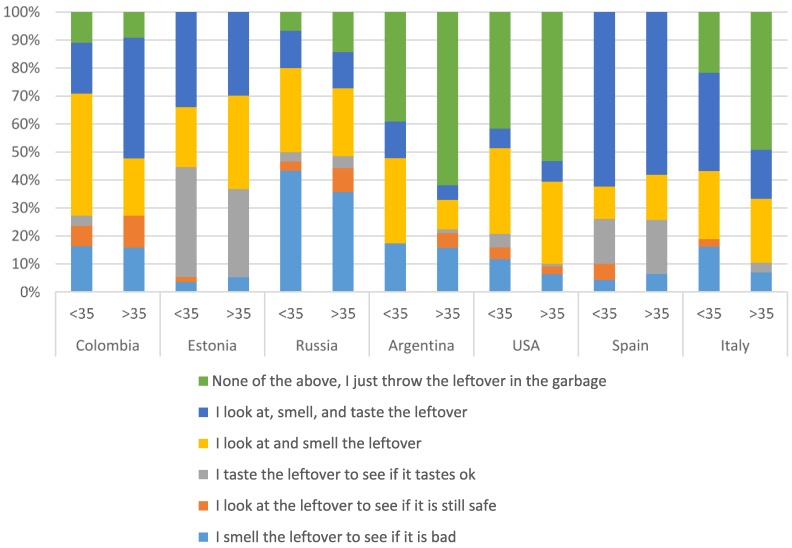
If you find a leftover in the refrigerator and do not know how long it has been in there, how do you determine if it is still safe to eat? (Select one). Results presented for respondents aged <35 and ≥35 in each country, by percentage.

Respondents were asked how they determined whether a leftover is still safe to eat when they did not know how long it had been stored in the refrigerator. In the United States (47%), Argentina (58%), Italy (38%), and Spain (61%), the majority of respondents indicated that when not knowing how long the leftover had been stored, they just threw it in the garbage without trying to determine if it could be eaten or not (Table 7). In Colombia (10%) and Russia (12%), this percentage was much lower. In Russia, most of the consumers (38%) answered that they smelled the leftover to determine if it was bad. In Estonia (35%) and Colombia (33%), most of the consumers looked at and smelled the leftover to determine if the leftover could still be consumed. Surprisingly, 22%–29% of the respondents in Estonia, Italy, and Colombia indicated that they looked, smelled, and tasted the leftover to determine safety. This could place many consumers at risk for foodborne illnesses. The practices were similar by age, except for Colombia, where younger people tended to look, smell, and taste the leftover before deciding if it was safe to consume (Figure 5).

## 4. Discussion

The results on behavior of leftover holiday meals presented some interesting results. The majority of the respondents stored the leftovers in less than two hours, as suggested by FSIS [17]. However, the results also show at least 15%–20% exhibiting problem behaviors (storage after more than two hours) in Italy, Spain, and the United States and over 60% admitting this in Estonia. That suggests this problem behavior is still common around the world, but much more common in some locations than others. This behavior can pose risks from a food safety standpoint of view, but it is likely difficult to change from a social standpoint of view. Holiday meals are typically social gatherings. Clearing the table of dishes can indicate the end of the eating occasion and, at the same time, social occasion. It would be interesting, for future studies, to look deeper into these situations to determine the type of interventions or guidelines needed to change the consumer behavior.

Leftover storage results indicated that more than half of the respondents in all of the countries studied used special containers to store leftovers from meals. Bruhn et al. found that two-thirds of the participants in their study used small containers to store food, while the remaining one-third preferred to use containers in which the meal was prepared [7]. Smaller containers are preferred over larger ones, in order to allow food to cool faster in the refrigerator. The United States Food Safety and Inspection Service (FSIS) suggests that consumers wrap leftovers or store leftovers in special containers [17]. These were the most common ways to store leftovers for the participants in this study in all of the countries. However, it should be noted that 9.2% of the United States, 11.0% of Russian, 15.2% of Colombian, and 16.4% of Estonian respondents indicated they stored the leftovers in the container in which the food was cooked in, and this could potentially cause food safety issues. Thus, work related to increasing consumer awareness of this necessary practice should continue.

The respondents were asked to indicate how they determined if the leftover was still safe to eat. Large portions of the consumers looked and smelled the leftovers. Surprisingly, large proportions of the respondents answered that they “looked, smelled, and tasted the leftovers”. A total of 6.9% of the United States respondents, 7.1% of Argentinian, 13% of Russian, 14% of Spanish, 24.5% of Italian, 27.4% of Estonian, and 29.3% of Colombian consumers indicated that they used most of their senses to determine leftover safety. Tasting leftovers to determine if they are spoiled is a potentially risky practice because the leftover being tasted may already be spoiled. Consumer guidelines do not actually indicate anything about determining safety of the leftovers using senses, including tasting. This may be an area for future studies to further investigate.

Respondents were asked how long they thought certain leftovers; such as meat dishes, vegetable dishes, fresh salads, cooked pasta, desserts, fruits, and restaurant dishes, can stay in the refrigerator before spoiling. Marklinder et al. found that leftovers were kept for a maximum of three days [1], and FSIS suggests for consumers to store leftovers for 3–4 days [17]. Kosa et al. reported consumer refrigeration practices in the United States; showing that owning refrigerator thermometers and keeping the refrigerators clean and at the correct temperatures, were practices that also varied in subpopulations [18]. In another country, Italy, the researchers found that 21.5% of the refrigerators were not holding the correct storage temperature, even though the consumers knew what the temperature should be [19]. Most participants in this study indicated that they thought meat dishes and restaurant dishes can be stored for 3–4 days in the refrigerator (Table 6). A total of 12.4% of the United States, 5.1% Colombian, 5% of Argentinian, and 5% of Russian respondents indicated they thought that meat dishes can be stored for up to a week. A total of 32% of Argentinians, 26% of Russians, 15% of Estonians, and 23.9% of Italian respondents selected the “do not know” option for storage of restaurant leftovers. This indicates some potentially risky practices when it comes to meat dishes, storage of restaurant dishes, and later usage. This may be related to the typical frequency of eating at restaurants. However, one thing to point out is that in the United States, the consumer is provided with a lot of food safety related information, such as guidelines from the FSIS, and different outreach programs. In other countries, this may or may not be the case, depending on resources available for consumer education and food safety-related research. Of course, these may come in different languages. For example, in Russia and Spain, some guidelines exist; though consumer education programs are scarce [20,21]. Thus; this study is not only looking at specific consumer behaviors and knowledge on food safety, but it is also pointing out that there are considerable food safety-related educational materials available that could be adopted in different countries. The approach to sharing all of these materials is yet to be determined and up for discussion.

Naturally, this study comes with limitations. Key among them is the limited sample size of the populations in each country, which cannot possibly represent all the consumers in that country. We do not suggest this is an exhaustive study, but rather should be viewed as a first look at cross-countries similarities and differences among consumer behaviors related to leftover foods. In addition, there are many socioeconomic and cultural differences among these countries that can have an enormous impact on behaviors. This research is not intended to be a study of those cultural impacts nor to identify particular cultural norms. Rather, it was the intent to simply describe the results of the study in terms of potential differences among countries and generalized sociodemographic behaviors. Further research is needed to study particular social or cultural influences on food safety behaviors.

## 5. Conclusions

This study focused on how consumers handled leftover foods in different countries: the United States, Argentina, Colombia, Estonia, Italy, Spain, and Russia. Several similarities were found, such as preference of choosing special containers for storing leftovers (by the majority of respondents), and a majority of women being the ones that usually stored leftovers more often than men. Further, most participants indicated they stored leftovers for a time period that is similar to the guidelines (3–4 days). However, some risky behaviors were identified in all countries; such as the time that food is left out before being stored, and storing certain types of leftovers for several days longer than they should be kept (in some cases up to a week). Of considerable concern is that a portion of consumers were tasting leftovers to determine safety—a risky practice. Those areas of concern appear to be the weak points that need further assessment for education within individual countries and worldwide.

## Figures and Tables

**Table 1:** Participant demographics in percentages by country.

Demographic Segmentation %	USA (*n* = 239)	Argentina (*n* = 100)	Colombia (*n* = 99)	Estonia (*n* = 113)	Italy (*n* = 94)	Russia (*n* = 100)	Spain (*n* = 99)
**Gender**							
Male	26.9	0.0	32.3	15.0	16.1	14.0	51.5
Female	73.1	100.0	67.7	85.0	83.9	86.0	48.5
**Age**							
18–34	56.3	23.0	53.0	8.0	39.4	30.0	39.4
≥35	43.7	77.0	46.0	92.0	60.6	70.0	60.6
**Education**							
Less than college	11.4	59.0	72.0	8.0	17.0	17.0	8.1
Some college courses or more	88.6	41.0	27.0	92.0	83.0	83.0	91.9
**Income**							
Low	24.7	52.0	90.9	64.6	22.5	19.0	23.7
Medium	26.4	27.0	8.1	31.0	52.8	67.0	42.3
High	48.9	21.0	1.0	4.4	24.7	14.0	34.0

**Table 2:** When you cook at home, do you usually save leftovers? Results given in % of respondents.

Answers:	USA	Colombia	Argentina	Estonia	Italy	Spain	Russia	*p*-Value
Always	49.6 *	**21.2**	**47.0**	**77.0**	**59.1**	**42.4**	**41.0**	0.000
Sometimes	**49.1**	**64.6**	**50.0**	**22.1**	**40.9**	**52.5**	**53.0**	0.001
Never	**1.3**	**13.1**	**3.0**	**0.9**	**0.0**	**5.1**	**6.0**	0.000
I do not cook at home	0.0	1.0	0.0	0.0	0.0	0.0	0.0	0.276
*p*-value (overall)								0.000

* Values shown in bold are statistically significantly different among the countries according to chi-square test (*p* < 0.05).

**Table 3:** If someone were to look in your refrigerator each day for a week; how many of the seven days do you think that they would find leftover food in your refrigerator? Results given in % of respondents.

Answers:	USA	Colombia	Argentina	Estonia	Italy	Spain	Russia	*p*-Value
None	**2.1**	**9.1**	**10.0**	**0.9**	**2.1**	**6.1**	7.0 *	0.003
One	**15.8**	**28.3**	**46.0**	**9.7**	**35.1**	**39.4**	**50.0**	0.000
Two or three	47.9	47.5	37.0	56.6	45.7	42.4	31.0	0.122
Four to six	**18.8**	**10.1**	**5.0**	**26.5**	**12.8**	**9.1**	**5.0**	0.000
All seven	**15.4**	**5.1**	**2.0**	**6.2**	**4.3**	**3.0**	**7.0**	0.000
*p*-value (overall)								0.000

* Values shown in bold are statistically significantly different among the countries according to chi-square test (*p* < 0.05).

**Table 4:** You have prepared food for a holiday meal and there is food left over. How long do you generally leave it out at room temperature before putting in the refrigerator? Results given in % of respondents.

Answers:	USA	Colombia	Argentina	Estonia	Italy	Spain	Russia	*p*-Value
Within half an hour	**14.2**	**9.1**	**30.7**	**4.4**	**22.3**	**41.8**	34.0 *	0.000
Within one hour	**36.9**	**25.3**	**23.8**	**8.0**	**31.9**	**26.5**	**17.0**	0.000
Within two hours	**25.3**	**39.4**	**8.9**	**19.5**	**29.8**	**14.3**	**12.0**	0.000
Within three to four hours	12.0	15.2	5.9	11.5	6.4	8.2	11.0	0.367
If people are still there, I let it sit until they leave, which may be many hours later	**6.9**	**8.1**	**24.8**	**55.8**	**8.5**	**7.1**	**25.0**	0.000
I never store leftovers in the refrigerator	0.4	2.0	1.0	0.0	1.1	2.0	1.0	0.831
I never cook a holiday meal	**4.3**	**1.0**	**5.0**	**0.9**	**0.0**	**0.0**	**0.0**	0.003
*p*-value (overall)								0.000

* Values shown in bold are statistically significantly different among the countries according to chi-square test (*p* < 0.05).

**Table 5:** How do you usually store leftover food in the refrigerator? (Check all that apply.) Results given in % of respondents.

Answers:	USA	Colombia	Argentina	Estonia	Italy	Spain	Russia	*p*-Value
In the container in which was cooked	**9.2**	**15.2**	**3.9**	**16.4**	**2.5**	**7.3**	11.0 *	0.000
In the container in which was served	**11.2**	**20.7**	**17.2**	**25.8**	**9.2**	**14.6**	**21.0**	0.000
In containers I have especially for leftovers	**60.7**	**41.5**	**60.9**	**36.4**	**62.2**	**64.2**	**49.0**	0.000
In plastic or aluminum wrap	**18.9**	**22.6**	**18.0**	**21.3**	**26.1**	**13.8**	**19.0**	0.000
*p*-value (overall)								0.000

* Values shown in bold are statistically significantly different among the countries according to chi-square test (*p* < 0.05).

**Table 6:** How long do you think certain leftovers can stay in the refrigerator before spoiling? (Check one box from each row). Results given in % of respondents.

Answers:		USA	Colombia	Argentina	Estonia	Italy	Spain	Russia	*p*-Value
Meat	1–2 days	**40.3**	**67.7**	**71.0**	**46.9**	**76.1**	**60.2**	58.0 *	0.000
	3–4 days	**45.5**	**27.3**	**20.0**	**50.4**	**22.8**	**37.8**	**36.0**	0.000
	1 week	**12.4**	**5.1**	**5.0**	**2.7**	**1.1**	**2.0**	**5.0**	0.000
	Do not know	**1.7**	**0.0**	**4.0**	**0.0**	**0.0**	**0.0**	**1.0**	0.040
	Does not matter	**0.0**	**0.0**	**0.0**	**0.0**	**0.0**	**0.0**	**0.0**	-
*p*-value (overall)									0.000
Vegetables	1–2 days	**27.9**	**77.8**	**76.0**	**35.4**	**62.8**	**67.7**	**57.0**	0.000
	3–4 days	**50.6**	**17.2**	**17.0**	**57.5**	**34.0**	**25.3**	**37.0**	0.000
	1 week	**17.6**	**5.1**	**0.0**	**7.1**	**3.2**	**6.1**	**6.0**	0.000
	Do not know	**0.9**	**0.0**	**7.0**	**0.0**	**0.0**	**1.0**	**0.0**	0.007
	Does not matter	3.0	0.0	0.0	0.0	0.0	0.0	0.0	0.601
*p*-value (overall)									0.000
Fresh Salad	1–2 days	**60.9**	**78.8**	**87.0**	**87.6**	**79.6**	**89.9**	**83.0**	0.040
	3–4 days	**29.2**	**17.2**	**6.0**	**8.0**	**16.1**	**9.1**	**15.0**	0.000
	1 week	6.4	4.0	1.0	3.5	3.2	1.0	2.0	0.133
	Do not know	**1.3**	**0.0**	**6.0**	**0.9**	**1.1**	**0.0**	**0.0**	0.043
	Does not matter	**2.1**	**0.0**	**0.0**	**0.0**	**0.0**	**0.0**	**0.0**	0.002
*p*-value (overall)									0.000
Cooked pasta	1–2 days	**23.8**	**46.5**	**61.0**	**30.1**	**60.2**	**42.9**	**46.0**	0.00
	3–4 days	42.4	48.5	32.0	51.3	34.4	52.0	37.0	0.150
	1 week	**29.0**	**5.1**	**3.0**	**17.7**	**3.2**	**4.1**	**14.0**	0.000
	Do not know	2.6	0.0	4.0	0.9	1.1	1.0	2.0	0.374
	Does not matter	2.2	0.0	0.0	0.0	1.1	0.0	1.0	0.212
*p*-value (overall)									0.000
Dessert	1–2 days	**14.7**	**48.5**	**31.0**	**36.3**	**47.3**	**45.5**	**66.0**	0.000
	3–4 days	**37.5**	**37.4**	**49.0**	**50.4**	**48.4**	**46.5**	**25.0**	0.041
	1 week	**42.2**	**11.1**	**17.0**	**9.7**	**4.4**	**8.1**	**7.0**	0.000
	Do not know	3.0	3.0	1.0	3.5	0.0	0.0	2.0	0.054
	Does not matter	2.6	0.0	2.0	0.0	0.0	0.0	0.0	0.296
*p*-value (overall)									0.000
Fruit	1–2 days	**19.0**	**5.1**	**9.0**	**9.7**	**21.3**	**20.2**	**23.0**	0.002
	3–4 days	**31.6**	**18.2**	**45.0**	**30.1**	**43.6**	**22.2**	**31.0**	0.004
	1 week	**41.1**	**76.8**	**44.0**	**54.9**	**31.9**	**53.5**	**27.0**	0.000
	Do not know	**6.1**	**0.0**	**2.0**	**2.7**	**2.1**	**4.0**	**17.0**	0.000
	Does not matter	2.2	0.0	0.0	2.7	1.1	0.0	2.0	0.308
*p*-value (overall)									0.000
Restaurant dishes	1–2 days	68.8	90.9	58.0	75.2	72.7	75.8	62.0	0.135
	3–4 days	**25.1**	**6.1**	**4.0**	**8.8**	**3.4**	**14.1**	**10.0**	0.000
	1 week	**3.5**	**3.0**	**3.0**	**0.9**	**0.0**	**1.0**	**2.0**	0.000
	Do not know	**2.6**	**0.0**	**32.0**	**15.0**	**23.9**	**9.1**	**26.0**	0.000
	Does not matter	**0.0**	**0.0**	**3.0**	**0.0**	**0.0**	**0.0**	**0.0**	0.000
*p*-value (overall)									0.000

* Values shown in bold are statistically significantly different among the countries according to chi-square test (*p* < 0.05).

**Table 7:** If you find a leftover in the refrigerator and do not know how long it has been in there, how do you determine if it is still safe to eat? Results given in % of respondents.

Answers:	USA	Colombia	Argentina	Estonia	Italy	Spain	Russia	*p*-Value
I smell the leftover	**8.9**	**16.2**	**15.2**	**4.4**	**10.6**	**5.0**	38.0 *	0.000
I look at the leftover	**3.7**	**9.1**	**4.0**	**0.9**	**1.1**	**4.0**	**7.0**	0.047
I taste the leftover	2.8	2.0	1.0	0.0	2.1	0.0	4.0	0.247
I look at and smell the leftover	**30.5**	**33.3**	**15.2**	**35.4**	**23.4**	**16.0**	**26.0**	0.017
I look at, smell, and taste the leftover	**6.9**	**29.3**	**7.1**	**27.4**	**24.5**	**14.0**	**13.0**	0.000
None of the above	**47.2**	**10.1**	**57.5**	**31.9**	**38.3**	**61.0**	**12.0**	0.000
*p*-value (overall)								0.000

* Values shown in bold are statistically significantly different among the countries according to chi-square test (*p* < 0.05).
